# Euglycemic diabetic ketoacidosis after the initiation of dulaglutide in patient with type 2 diabetes

**DOI:** 10.1097/MD.0000000000034027

**Published:** 2023-06-09

**Authors:** Rabia Khalid Alduraibi, Yazeed Mohammed Alrebdi, Yosef Fahad Altowayan

**Affiliations:** a Department of Endocrine and Diabetes, King Fahad Specialist Hospital, Buraydah, Saudi Arabia; b Department of Internal Medicine, King Fahad Specialist Hospital, Buraydah, Saudi Arabia.

**Keywords:** diabetic ketoacidosis, euglycemic, GLP1 receptor agonists, SGLT2 inhibitors, type 2 diabetes

## Abstract

Diabetic ketoacidosis is rarely observed when the blood glucose level is <250 mg/dL. This is referred to as euglycemic diabetic ketoacidosis (EDKA). EDKA can present diagnostic and management challenges for physicians, especially when dealing with unusual triggers such as glucagon-like peptide 1 (GLP1) receptor agonists and sodium-glucose co-transporter 2 inhibitors. With this case report, we wanted to raise the knowledge and understanding of EDKA and its triggering factors.

**Patient concerns:**

A 45-year-old man was admitted to hospital for epigastric pain, loss of appetite, and vomiting 3 days after the initiation of dulaglutide. The results of laboratory examination showed EDKA.

**Diagnoses:**

The patient was diagnosed with EDKA after the initiation of GLP1 receptor agonists.

**Interventions:**

Intravenous fluid and insulin infusion were immediately started.

**Outcome:**

The patient was discharged after treatment

**Lessons:**

In this case report describes the use of GLP1 receptor agonists along with Sodium-glucose co-transporter 2 inhibitors in type 2 diabetes patients whose extreme restriction of carbohydrate intake may have triggered EDKA. Therefore, physicians should use diabetes medications in a stepwise manner and advise their patients not to over-restrict their carbohydrate intake while they are being treated with GLP1 receptor agonists.

## 1. Introduction

Diabetic ketoacidosis (DKA) is the hallmark of a potentially fatal medical emergency in patients with type 1 diabetes who are newly diagnosed or whose disease is poorly managed. This traditional association has been challenged in recent decades by the rising number of reports of patients with type 2 diabetes presenting with DKA.

DKA is rarely observed when blood glucose (BG) levels are < 250 mg/dL. When it is, this condition is known as euglycemic diabetic ketoacidosis (EDKA). EDKA is characterized by metabolic acidosis (pH < 7.3), a serum bicarbonate level < 18 mEq/L, and positive ketones in the serum and urine all in the presence of BG level is <250 mg/dL.^[1]^

The possible causes of EDKA include decreased food consumption with persistent vomiting, pancreatitis, sepsis, and alcoholic ketoacidosis.^[2,3]^

It has also been reported that sodium-glucose co-transporter 2 (SGLT2) inhibitors raise the risk of EDKA in patients with type 2 diabetes regardless of the duration of exposure.^[4]^

This group of relatively new drugs has been shown to enhance ketone reabsorption by the kidneys, increase glucose excretion and inhibit its reabsorption, and promote the release of glucagon. These changes can lead to a condition of carbohydrate insufficiency, more fluid loss, an increased ratio of glucagon to insulin, more lipolysis, and ketosis.^[5–7]^ In addition, the gastrointestinal side effects of glucagon-like peptide 1 (GLP1) receptor agonists, as well as energy restriction, may predispose patients to DKA, as in our patient.

Here, we report a case of EDKA in a type 2 diabetic patient who recently changed his diabetes medications and started on GLP1 receptor agonists (dulaglutide) along with metformin, gliclazide, and empagliflozin. This case presents an opportunity to discuss how to recognize and manage this challenging condition.

To our knowledge, this is the first report discussing the risk of EDKA with the use of GLP1 receptor agonists together with SGLT2 inhibitors and the extreme restriction of carbohydrate intake in type 2 diabetes patients in Saudi Arabia.

## 2. Case presentation

A 45-year-old man presented to our emergency department with a 3-day history of a loss of appetite, abdominal pain, nausea, and continuous bilious, non-bloody vomiting after he received his first dose of dulaglutide (GLP1 receptor agonist) 1.5 mg subcutaneous injections 3 days before coming to the hospital. The pain was epigastric, 7/10 in intensity, progressive, and non-radiating. The patient denied fever, cough, chest pain, or shortness of breath.

The patient had been diagnosed with type 2 diabetes for 8 years and had been treated with gliclazide 120 mg, metformin 1000 mg, and empagliflozin 25 mg daily for the past 5 years. He reported adherence to medication, and the last dose taken of all his medication was 2 days prior to admission. He had been on a low-carbohydrate diet for 2 months, and it was the only noticeable change in his routine.

In admission he was conscious, alert, and oriented. His Glasgow Coma Scale score was15. Vital signs consisted of pulse of 99 beats/minute, temperature of 36.8°C, a respiratory rate of 22 breaths/minute, and a blood pressure of 110/75 mm Hg. Abdomen was soft, and non-tender. The remainder of the physical examination was normal.

The initial laboratory test revealed severe EDKA. His arterial pH was 6.95, his serum bicarbonate level was 4 mmol/L, his pCO2 was 28 mm Hg, his anion gap was 32, and his BG level was 240 mg/dL. His HbA1c was 10.8%, hemoglobin was 15.3 g/day, and white blood cell count was 16.2/μ. Serum sodium was 142 mEq/L, potassium was 5 mmol/L, chloride was 103 mEq/L, triglycerides were 0.4 mmol/L, amylase was 36 U/L. Serum C peptide was 0.7 nmol/L (0.37–1.47), with negative GAD and anti-IA2 autoantibodies. The dipstick urine test revealed the presence of 3 + ketones and 3 + glucose. Furthermore, he underwent an abdominal ultrasound, the findings of which were normal. EDKA was confirmed as a diagnosis most likely precipitated by GLP1 receptor agonists; Other etiologies, such as infection and metformin-induced lactic acidosis, were ruled out with additional testing, which revealed a lactic acid level of 1.5 mmol/L (normal 0–2), an ethanol level of, 8 mg/dL, and a negative salicylate level. A chest X-ray revealed no signs of pneumonia, and urinalysis results were unremarkable.

Intravenous fluid and insulin were immediately started. His DKA responded within 24 hours of this medical management. The patient was sent home on a basal bolus insulin regimen.

## 3. Discussion

EDKA is potentially fatal complication of diabetes that can present in both type 1 and type 2 diabetes mellitus patients. EDKA is characterized by metabolic acidosis (pH <7.3), a serum bicarbonate level < 18 mEq/L, and positive ketones in the serum and urine all in the presence of BG level is <250 mg/dl.^[5]^

in 1973, EDKA was defined by Munro et al^[8]^ as a discrete entity. He reported a group of 211 patients with DKA, 37 of whom had BG levels <300 mg/dL at presentation; all of these patients were initially diagnosed with type 1 diabetes.

In the 1980s and 1990s, larger epidemiologic studies were conducted on EDKA. It was found that the incidence of EDKA is between 1% and 3.2% of patients presenting with DKA, which suggests that it is a rare condition.^[9]^

The development of EDKA has been associated with several precipitating factors, such as extreme restriction of carbohydrate intake, pancreatitis, sepsis, pregnancy, and toxic alcohol consumption.^[2,3]^ The absence of clear precipitating factors may make EDKA diagnosis challenging and can easily lead to misdiagnosis.

To the best of our knowledge, this is the first case report in Saudi Arabia discussing the risk of EDKA associated with the use of GLP1 receptor agonists together with SGLT2 inhibitors and the extreme restriction of carbohydrate intake in patients with type 2 diabetes. EDKA can be challenging to diagnose due to a variety of factors. EDKA has become more common in type 2 diabetes patients once the introduction of SGLT2 inhibitors. These newer drug groups work by increasing glucose excretion at the kidney level, inhibiting its reabsorption, and enhancing ketone reabsorption by the kidney.

in June 2019, the UK Medicines and Healthcare Products Regulatory Agency reported that DKA may occur in type 2 diabetes patients receiving combined GLP1 receptor agonists and insulin therapies when insulin is abruptly reduced or discontinued.^[10]^

Likewise, in December 2019, the FDA Adverse Event Reporting System reported that the DKA risk associated with GLP1 receptor agonists needed to be assessed.^[11]^

However, GLP1 receptor agonists have become an important group of antidiabetic drugs in management of type 2 diabetes. This relatively new group of drugs has the following effects: improved insulin release, slow gastric emptying and decrease appetite.^[12]^

The American Diabetes Association also recommended that GLP1 receptor agonists should be one of the first combination drugs considered in the treatment of patients with type 2 diabetes who have developed atherosclerotic cardiovascular disease or high-risk indicators of Atherosclerotic cardiovascular disease.^[13]^

GLP1 receptor agonist-related side effects such as gastrointestinal symptoms,^[14]^ pancreatitis,^[15]^ thyroid disease and diabetic retinopathy.^[16]^

There is a case report describing DKA in type 2 diabetes patients after stopping insulin and initiating dulaglutide therapy.^[17]^ Recently, no large sample study has been conducted to demonstrate the relationship between GLP1 receptor agonists and DKA, and this remains unknown.

In our patient, the Initiating treatment with GLP1 receptor agonists with extreme restriction in carbohydrate intake could have resulted in increased lipolysis, impaired insulin, and increase glucagon release, resulting in EDKA within days. Therefore, physicians should caution patients not to extreme restrict carbohydrate intake while on GLP1 receptor agonist therapy.

## 4. Conclusion

In conclusion, this case report describes the use of GLP1 receptor agonists along with SGLT2 inhibitors in type 2 diabetes patients whose extreme restriction of carbohydrate intake may have triggered EDKA. Therefore, physicians should use diabetes medications in a stepwise manner and advise their patients not to over-restrict their carbohydrate intake while they are being treated with GLP1 receptor agonists.

## Acknowledgements

We are grateful to the patient and her family who kindly consented to participate in the study.

## Author contributions

**Conceptualization:** Rabia Khalid Alduraibi.

**Writing – original draft:** Rabia Khalid Alduraibi, Yazeed Mohammed Alrebdi, Yosef Fahad Altowayan.

**Writing – review & editing:** Rabia Khalid Alduraibi, Yazeed Mohammed Alrebdi, Yosef Fahad Altowayan.
